# Characterization of Chromatic Dispersion and Refractive Index of Polymer Optical Fibers

**DOI:** 10.3390/polym9120730

**Published:** 2017-12-20

**Authors:** Igor Ayesta, Joseba Zubia, Jon Arrue, María Asunción Illarramendi, Mikel Azkune

**Affiliations:** 1 Department of Applied Mathematics, Engineering School of Bilbao, University of the Basque Country (UPV/EHU), Plaza Ingeniero Torres Quevedo, 1, E-48013 Bilbao, Spain; 2 Department of Communications Engineering, Engineering School of Bilbao, University of the Basque Country (UPV/EHU), Plaza Ingeniero Torres Quevedo, 1, E-48013 Bilbao, Spain; joseba.zubia@ehu.eus (J.Z.); jon.arrue@ehu.eus (J.A.); mikel.azkune@ehu.eus (M.A.); 3 Department of Applied Physics I, University of the Basque Country (UPV/EHU), Engineering School of Bilbao, Plaza Ingeniero Torres Quevedo, 1, E-48013 Bilbao, Spain; ma.illarramendi@ehu.eus

**Keywords:** polymer optical fiber, refractive index, chromatic dispersion, poly(methyl methacrylate), Streak Camera, communications links

## Abstract

The chromatic dispersion and the refractive index of poly(methyl methacrylate) polymer optical fibers (POFs) have been characterized in this work by using a tunable femtosecond laser and a Streak Camera. The characterization technique is based on the measurement of the time delays of light pulses propagating along POFs at different wavelengths. Polymer fibers of three different lengths made by two manufacturers have been employed for that purpose, and discrepancies lower than 3% have been obtained in all cases.

## 1. Introduction

Due to their flexibility and ease of use, poly(methyl methacrylate) (PMMA) polymer optical fibers (POFs) have very interesting characteristics for short-haul communications links, as well as for other applications in fields such as optical sensing, illumination, and light amplification and lasing [1,2,3,4,5]. In most POF communications links, on-off-keying direct modulation of the optical source (laser or light-emitting diode (LED)) is employed. Owing to dispersion, a light pulse is usually broader in time at the output fiber end than at the input one. Dispersion seriously limits the transmission capacity of POFs as a consequence of pulse overlapping, which complicates signal detection and, consequently, increases the bit error rate [6]. Pulse broadening along the POF is mainly caused by two dispersion mechanisms, namely, intermodal dispersion (also known as modal) and intramodal dispersion (also known as chromatic). There is a third dispersion mechanism called polarization-mode dispersion, but it can usually be ignored in POFs [7]. As for modal dispersion, it is the different paths that light can follow along the fiber core that produce the pulse widening. On the other hand, chromatic dispersion is caused by the nonzero spectral width of the light source. It comprises both material and waveguide dispersion, but the latter is negligible in POFs [7]. Material dispersion, and chromatic dispersion in general, only depends on the core material and not on the type of fiber, i.e., step-index (SI) or graded-index (GI). It is produced by the wavelength dependence of the core refractive index [8]. Although this type of dispersion is typically smaller than the modal one, we cannot neglect it, especially in the case of GI POFs, whose use for communications links has spread significantly in the last few years [9]. Therefore, it is becoming increasingly important to characterize both the chromatic dispersion and the refractive index of POFs in the range of wavelengths that are normally used. In other applications, a detailed knowledge of the dispersion properties is also required. This happens, for instance, in the field of spectroscopy. As an example, POFs, and highly dispersive fibers in general, could be utilized to convert short broadband optical pulses into fast wavelength scans [10].

There are three principal techniques in the literature to characterize the chromatic dispersion of optical fibers: interferometric, phase-shift, and time-of-flight [11]. Interferometric techniques require precise knowledge of the mechanical components employed in the set-up because slight deviations in the physical dimensions of the interferometer produce significant discrepancies in the measurement results. For this reason, the precision achievable with this method is very high in theory but not as high in practice, so the potential improvement in precision with respect to the other two methods is not so clear. Moreover, this technique is limited to very short fiber lengths. On the other hand, phase-shift techniques can be used with both short and long fiber lengths, but they require tunable light sources of known characteristics, since the phase shifts have to be determined from a sequence of individual wavelengths. A reference signal is usually used to overcome this problem. The precision of this technique depends on the stability of the tunable light sources, so it is not clear that its results are better than those obtainable with the other two techniques. Finally, the time-of-flight technique is comparatively easier to implement, due to the absence of a reference signal, its simplicity, and its speed of execution. Therefore, it is a very suitable technique for the measurement of the dispersion of optical fibers.

Although there are some works that have characterized the chromatic dispersion and the refractive index of optical fibers by means of these techniques, all of them have focused on glass optical fibers [11,12,13,14,15]. However, there are only a few works that characterize the dispersive behavior of polymer fibers and, if they do, they get the values from the bulk material or from the fiber preform, instead of from the POF itself [16,17,18]. In addition, these works usually require hand-made experimental measurements, apart from some time-consuming data analyses. In contrast, the fibers by themselves serve in our work to characterize the refractive index and the chromatic dispersion of POFs. As a matter of fact, all the characterizations of fiber dispersion in POFs reported in the literature were based on measurements using the preform, but the results in this paper have been obtained from the drawn fiber instead of the perform. For this purpose, the pulse delays are measured by temporally and spectrally resolving propagated pulses at different excitation wavelengths. The measurements, which are based on a time-of-flight approach, have been carried out by using a tunable laser as the excitation source and a Streak Camera as the measuring device. As far as we know, this is the first time that this kind of characterization has been carried out in POFs. Moreover, the measurement system can be widely automated, and the data analysis is straightforward, so the POF samples can be characterized in a very brief period of time.

This paper is organized as follows. First, the theoretical background is explained. Then, the experimental set-up is described, together with the characteristics of the optical fibers employed. Afterwards, the measuring techniques and the results are shown. Finally, the conclusions are summarized.

## 2. Theoretical Background

The refractive index of the material of the fiber core varies with the light wavelength *λ*. This variation can be approximated by the following power series, which is based on the Cauchy equation [19]:

(1) $$n\left(\lambda \right)=A+B\lambda^{-2}+C\lambda^{-4}+\ldots +M\lambda^{2}+N\lambda^{4}+\ldots ,$$

the constants *A*, *B*, *C*, *M*, *N*, … must be determined for the interval of wavelengths considered. Often, the first two terms are sufficient to represent the refractive index properly, which simplifies the expression to [19]:

(2) $$n\left(\lambda \right)=A+B\lambda^{-2},$$

The group-time delay undergone by a light pulse as it propagates through a POF of length *L* can be derived from Equation (2), obtaining the following expression for a pulse whose spectrum is centered at a certain wavelength *λ*:

(3) $$t_{g}\left(\lambda \right)=\frac{L}{c}\left[n\left(\lambda \right)-\lambda \frac{dn\left(\lambda \right)}{d\lambda }\right]=\frac{L}{c}(A+3B\lambda^{-2}),$$

where *c* is the speed of light.

Therefore, the corresponding material-dispersion coefficient is given by [20]:

(4) $$D\left(\lambda \right)\approx \frac{1}{L}\frac{dt_{g}}{d\lambda }=-\frac{\lambda }{c}\left(\frac{d^{2}n\left(\lambda \right)}{d\lambda^{2}}\right)=\frac{-6B\lambda^{-3}}{c},$$

In this expression, the symbol ≈ means that the equation is only valid when the interval of wavelengths considered (Δ*λ*) is small enough as to be able to use $\Delta t=\left(dt_{g}/d\lambda \right)\Delta \lambda$.

## 3. Experimental Set-Up

The various fibers analyzed in this work are commercially-available polymer optical fibers developed by Toray Industries Inc. and by Mitsubishi International PolymerTrade Corporation [21,22]. From each manufacturer, three different fiber lengths were prepared, namely, 25, 50, and 75 m. The fiber end-faces were carefully polished by hand using polishing papers. All the fibers employed are SI ones with high-purity PMMA cores and fluorinated-polymer claddings. Table 1 summarizes the main mechanical and optical characteristics of the fiber samples employed in this work.

**Figure 1:**
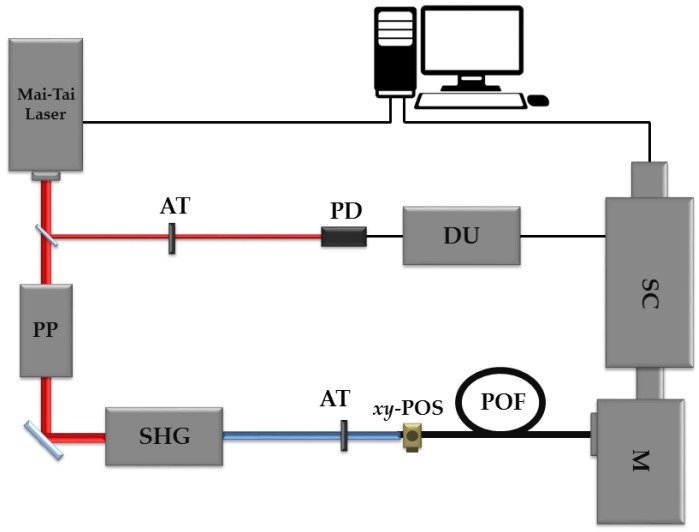
Experimental set-up employed to carry out the measurements. Legend: AT: variable attenuator, PD: photodetector, DU: delay unit, PP: pulse picker, SHG: second-harmonic generator, *xy*-POS: *xy*-micropositioner, POF: polymer optical fiber, M: monochromator, SC: Streak Camera.

Figure 1 sketches the experimental set-up employed to carry out our measurements. It includes a tunable ultrafast femtosecond laser (Spectra Physics Mai-Tai HP, Newport, Santa Clara, CA, USA) with a repetition rate of 80 MHz and a spectral emission range between 690 and 1040 nm. The selection of the desired emission wavelength is easily carried out by means of the specific software provided by the manufacturer. The laser emits pulsed beams of around 100 fs, 1.2 mm in diameter, and with a Gaussian-spatial distribution. The spectral width of the pulses is around 15 nm. For our measurements, the repetition rate of this laser was reduced to 8 MHz by using a pulse picker (Spectra Physics 3986, Newport, Santa Clara, CA, USA), which is based on an acoustic-optic modulator. In order to obtain excitation in the visible range from 400 to 520 nm, a second-harmonic generator (Inspire Blue, Radiantis, Barcelona, Spain) was employed. The impinging power is controlled by manually adjusting the variable attenuator placed before the POF sample. For all the wavelengths considered, the fibers were excited with an average light power of 9 nW, which is well below the limit that would produce damage in the fiber and nonlinear effects. The measured fiber samples were held by an *xy*-micropositioner in order to focus the incident laser beam just on the fiber-end face. The transmitted pulses were acquired by means of a combination of a monochromator (Acton SP2300, Princeton instruments, Trenton, NJ, USA) and an ultrafast Streak Camera (C10627, Hamamatsu, Hamamatsu City, Japan). This combination of both devices gives the option to resolve the acquired signals both spectrally and temporally, which means that the whole spectrum is obtained at the same instant of time. The maximum possible acquisition-wavelength range extends from 0 to 1400 nm. In order to properly synchronize both the excitation and the acquisition systems, a small amount of the laser signal is diverted from the laser output and directed to a photodetector, whose electric signal is delayed by means of a delay unit. A precise use of this delay unit guarantees the visualization of the transmitted pulses on the corresponding temporal window of the Streak Camera. The acquisition system is controlled by a 2.66 GHz Intel personal computer (PC) with 2 GB of random access memory (RAM). Both parts of the acquisition system and the measurements are controlled by the Hamamatsu HPDTA software (High-Performance Digital Temporal Analyzer, Hamamatsu, Hamamatsu City, Japan), which allows real-time data acquisition. Once the acquisition was performed, the data analysis was carried out by employing an ad-hoc Matlab program developed for this purpose.

On the other hand, we were able to carry out Raman measurements by using a confocal Raman spectrometer (InVia, Renishaw, Wotton-under-Edge, UK) at room temperature. The source in this case was a 532 nm laser operated at only 1% of the maximum emitting power in order to avoid any damage on the sample material. The spectra of the fiber cores can be measured by placing each of the samples parallel to the laser beams on the excitation plane, thus allowing us to visualize the core end-face in the Raman microscope and to excite the core material alone. On the other hand, the Raman bands of each of the claddings can be obtained by placing the sample perpendicular to the laser beams. However, due to the small thickness of the cladding, about 10 μm, the Raman equipment had to be set in its high-confocality mode before the experiment in order to excite the cladding material alone. In all cases, a 50× objective was employed and the acquisition time was 30 s.

## 4. Measuring Technique and Results

**Figure 2:**
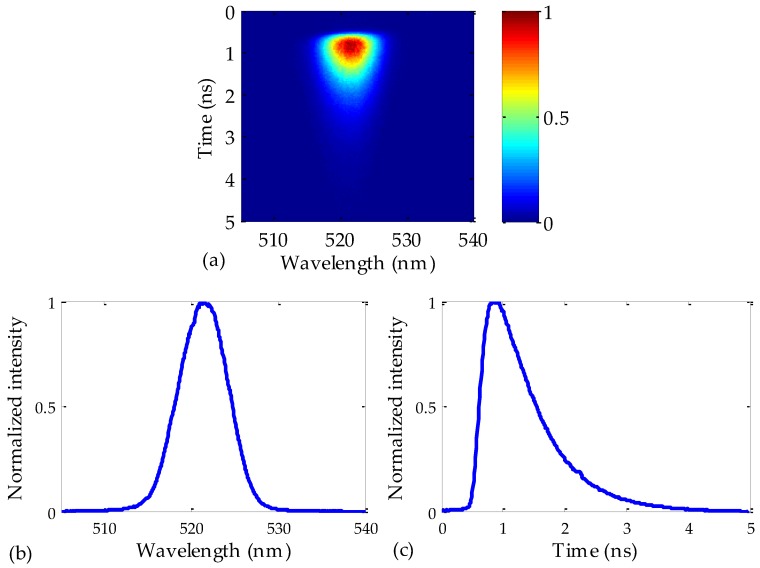
(**a**): Pseudo-3D Streak image obtained when a 50 m Toray fiber is excited at 520 nm with 9 nW of average power; (**b**,**c**): Respectively, spectral (time-integrated) and temporal (wavelength-integrated) images.

As an example of how the measurements were carried out, Figure 2a shows a Streak image obtained from the acquisition system. It corresponds to an excitation pulse centered at 520 nm that was launched into a 50 m Toray fiber. The spectral features of the analyzed image along the horizontal axis are limited by the monochromator, which includes three interchangeable gratings with grooves of different sizes, whereas the temporal features (vertical axis) are provided by the Streak Camera located after the monochromator. As shown in Figure 2a, the observed spectral span is 35 nm, which is given by the characteristics of the grating. In all of our measurements, a grating with 300 grooves/mm was employed. As for the temporal span, this was 5 ns, which is wide enough to capture the whole signal even if the longest fiber length is employed, namely, the 75 m fiber.

Figure 2b,c represent, respectively, the time-integrated and the wavelength-integrated images of Figure 2a. The spectral shape of the output pulse is almost symmetrical (a Gaussian-type curve), whereas its temporal shape is not. This asymmetry, which worsens when the fiber length is increased, is a consequence of the behavior of the modal dispersion, because the arrival of the fastest modes (the lowest-order ones) causes a rapid increase in the output intensity, but the intensity decreases gradually as the slowest modes (the highest-order ones) reach the output fiber end. This effect can constitute a serious issue when high-bit-rate communications links are needed.

Since one of our goals is to characterize the chromatic dispersion of POFs, it is enough to accurately determine the instants of maximum pulse intensity, without having to directly measure the temporal and spectral widths of the pulses. In this respect, the wavelength-integrated image obtained from Figure 2a can be employed to accurately calculate the instant of maximum intensity at the fiber output (see Figure 2c). Specifically, this value turns out to be 0.85 ± 0.01 ns in the figure. The process was repeated for different excitation wavelengths, i.e., the same fiber sample was excited from 400 to 520 nm, with steps of 15 nm. The relative time delays were calculated in two steps: first, the instant of the maximum was localized in the frame of the delay window employed to visualize the pulse, and then the relative delay of that frame window with respect to the previous one was taken into account, by means of the delay unit of the Streak Camera (see Figure 1). Due to the high degree of automatization in the excitation and in the measuring systems, the whole set of measurements was performed in just a few minutes. Once the relative time delay between different pulses was obtained, it was necessary to determine the absolute time delay when they propagated through the optical fiber. In order to do so, a 50 cm fiber was used as a reference to measure the time taken by the 520 nm pulse to reach the fiber output. By comparing the time delays in our reference fiber and in the sample by means of the delay unit of the camera, we determined the absolute arrival time of a pulse at the considered wavelength of 520 nm. Specifically, that value was 254.4 ns in the 50 m fiber. The delay of the rest of the pulses at other wavelengths were transformed into absolute values by taking into account the measured relative delays with respect to the pulse of 520 nm. It is worth mentioning that the maximum error when using a 50 cm fiber as a reference was only 1%, in the case of the 50 m sample.

**Figure 3:**
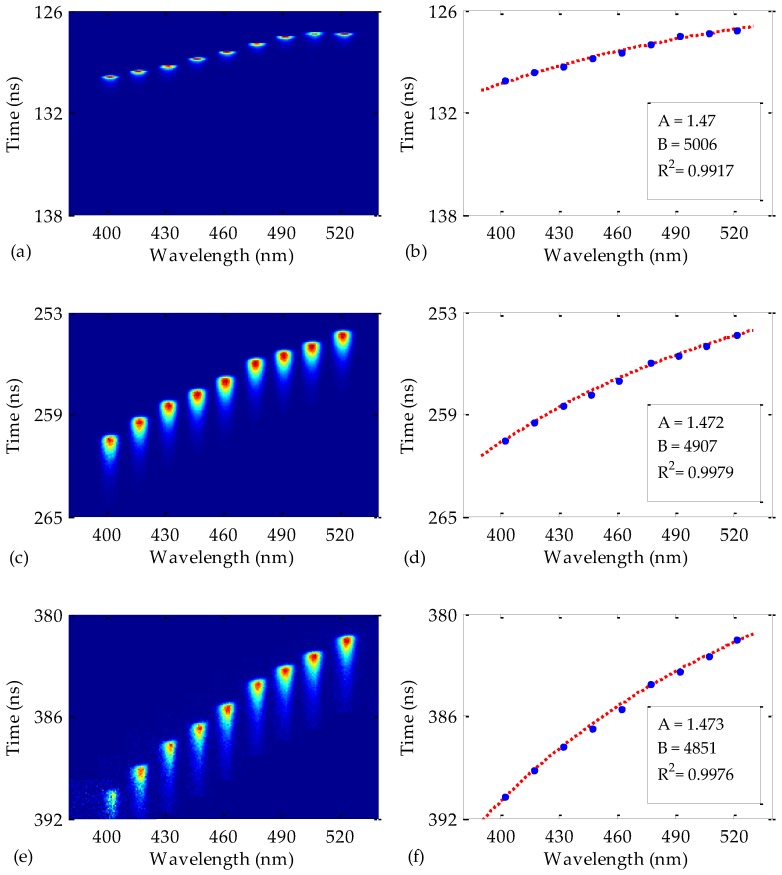
(**a**,**c**,**e**): Streak images of the output pulses measured from Toray fibers whose lengths are 25, 50, and 75 m, respectively. (**b**,**d**,**f**): corresponding instants of maximum pulse intensity together with the fittings to Equation (3). The insets show the resulting constants and the corresponding coefficients of determination of the fittings.

The fiber length employed for Figure 2 was 50 m, but the measurements were also repeated for other fiber lengths. The results for 25, 50, and 75 m are shown in Figure 3 for a train of pulses launched into each fiber (all the pulses of the train superimposed in the same figure by taking into account the absolute time delays obtained as described above). Notice that the first pulses that reach the fiber output end are those centered at longer wavelengths, at which the refractive index is lower [16]. The fiber dispersion is also noticeable in this figure, since the temporal width of the measured pulses widens as the fiber length is increased. On the other hand, it can be noticed that the pulses are affected by the PMMA attenuation, but not in the same way for all the pulses. The influence of this attenuation may be observed in the blurriness of the pulses of shorter wavelengths, i.e., of higher attenuation, as expected, this blurriness being more obvious as the fiber length is increased.

**Figure 4:**
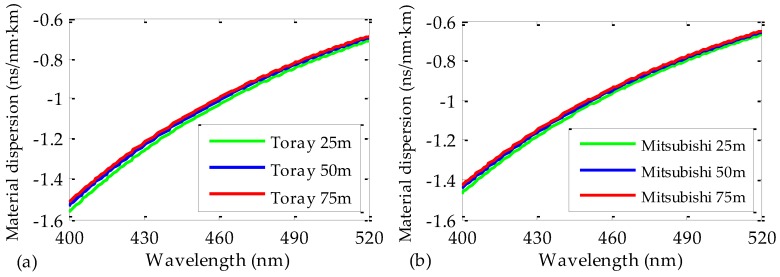
Material-dispersion coefficients obtained for POFs made by Toray (**a**) and by Mitsubishi (**b**). Three different lengths of fiber were employed for each manufacturer: 25 m (green lines), 50 m (blue lines), and 75 m (red lines).

The instants of maximum pulse intensity in Figure 3a,c,e are shown in the corresponding figures on the right-hand side. The least-squares fittings of these points to Equation (3) give us the expressions for the spectral time delays for each fiber length. The resulting constants for Equation (3) and the corresponding coefficients of determination of the fittings (*R*^2^) are summarized in Table 2. These constants are employed in Equation (4) to achieve the material dispersion coefficients *D*(*λ*) of the fibers. The results are shown in Figure 4a. It can be noticed that the value of *D*(*λ*) slightly depends on the fiber length. Specifically, the maximum relative discrepancy from one fiber length to another is around 3% at the wavelength interval considered in the figure. This means that the employed fiber length does not alter the results significantly when characterizing the dispersion of POFs.

Despite the high coefficients of determination in Table 2, one of them (corresponding to the 25 m Toray fiber) is lower than the other two; but this lower value could be improved by reducing the temporal window of the Streak Camera while maintaining the number of temporal pixels. That procedure would increase the temporal resolution. However, it would imply a more complex synchronization process, thus leading to a longer measurement time. For the choice of a more appropriate fiber length, it should also be taken into account that the 75 m fiber is affected by the detrimental effect of the higher attenuation on the quality of the pulses. In order to partially compensate for this loss of quality, we also tried to employ higher integration times. However, this implied longer measurement times and the improvement achieved was small. Consequently, the most suitable fiber length among the three considered was 50 m in the conditions employed for this work. In any case; it is worth mentioning that the optimum fiber length would depend on the excitation and measurement conditions.

**Figure 5:**
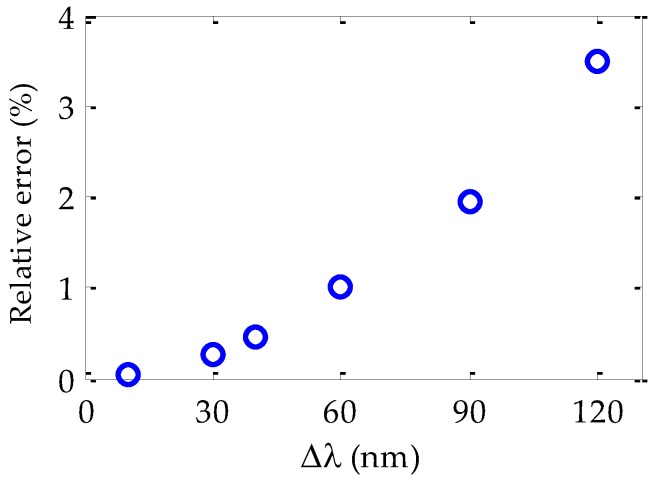
Relative error (%) obtained in the material dispersion coefficients as a function of the wavelength interval Δ*λ* for the 50 m Toray fiber.

It should be noted that the dispersion results were obtained by using Equation (4), which is only valid when the considered wavelength interval Δ*λ* is small, as was explained in Section 2. The lack of accuracy when Δ*λ* is too large can be observed in Figure 5, in which the results obtained by using Equation (4) for the 50 m Toray fiber are compared with the dispersion values corresponding to larger intervals of Δ*λ* (from 10 to 120 nm). The discrepancies are plotted as relative errors. In such intervals, the derivative (*dt*_g_/*dλ*) that is necessary to calculate *D*(*λ*) was estimated by using the two edges of the wavelength interval considered. As can be observed, the relative error is lower than 1% only when the wavelength interval is smaller than 40 nm. In other words, for any light source centered at any wavelength, the dispersion given by Equation (4) is accurate as long as the spectral width is not broader than 40 nm, taking the criterion of the 1%.

We also analyzed three PMMA fiber samples manufactured by a second manufacturer, i.e., Mitsubishi (see Table 1 for further details). The lengths of these fibers were, again, 25, 50, and 75 m. In this respect, the process was carried out under the same conditions and exactly in the same way as described above, and the obtained results for the material dispersion are shown in Figure 4b and Table 2. The new measurements using a different brand confirm again that the dispersion coefficients are independent of the fiber length. Moreover, for each fiber length, the results obtained with both brands of fiber (Toray and Mitsubishi) are almost identical, the maximum difference detected being 3%.

**Figure 6:**
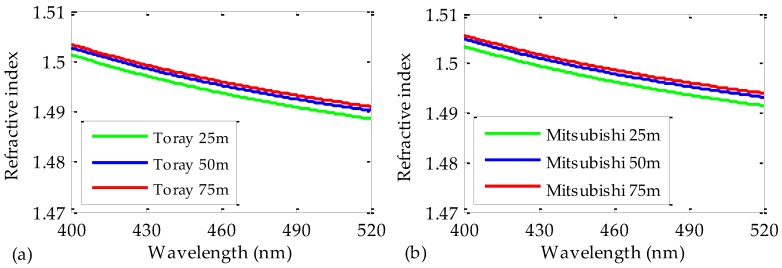
Refractive indices obtained for POFs made by Toray (**a**) and by Mitsubishi (**b**). Three different lengths of fiber have been employed for each manufacturer: 25 m (green lines), 50 m (blue lines), and 75 m (red lines).

Once the coefficients *A* and *B* of Equation (3) have been obtained, they can be introduced into Equation (2) in order to calculate the values of the refractive indices of the measured POFs. The results obtained by doing so for all the POF samples are shown in Figure 6. Again, the results are independent of the employed fiber length. Notice that the zoomed vertical scale in the figure causes the discrepancies to seem greater. However, the maximum relative errors are of only 0.13% and 0.2% for the Toray and the Mitsubishi fibers, respectively. Similarly, the results obtained for both manufacturers are alike again, the maximum difference between them being of 0.3% in the measured range of wavelengths.

**Figure 7:**
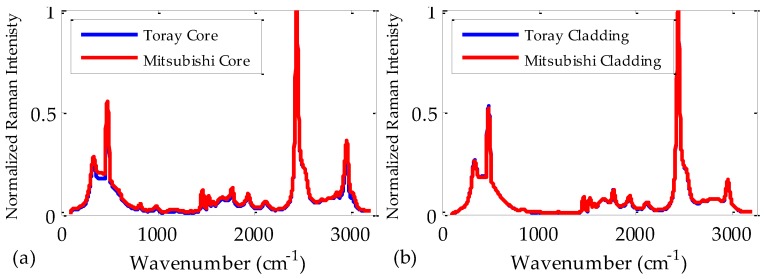
Normalized Raman spectrum measured for the cores (**a**) and for the claddings (**b**) of two different brands of fiber: Toray (blue lines) and Mitsubishi (red lines). With both manufacturers, the core is made of PMMA and the cladding is made of a fluorinated polymer.

All these results allow us to conclude that the most important issue affecting the refractive index and the chromatic dispersion is the fiber material. In order to corroborate this, we made some Raman measurements. In this respect, most of the Raman bands shown in Figure 7 coincide with those available in the literature [23,24]. At first glance, we can see in Figure 7a that the Raman bands of the fiber cores do not depend on the fiber manufacturer. We can also see that the same happens with the bands of the claddings, because the materials employed are the same in both cases. Consequently, it is reasonable to have obtained that both the refractive indices and the coefficients of chromatic dispersion in our POFs are almost independent of the manufacturer. Note, in Figure 7a, the slightly higher values obtained for the Mitsubishi fiber as compared to those of the Toray fiber. This effect can be a consequence of a more intense fluorescence emission in this fiber, which does not have any influence on its refractive index or its chromatic dispersion.

**Figure 8:**
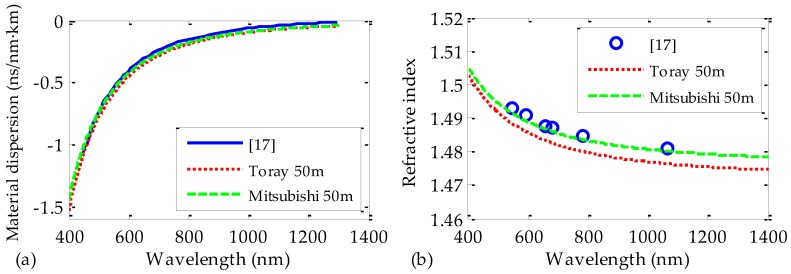
Coefficients of material dispersion (**a**) and refractive indices (**b**) obtained for both brands of fiber, together with the values reported by other authors from measurements of fiber preforms [17].

Finally, we compared the values obtained in this work for the refractive indices and for the coefficients of chromatic dispersion with those reported by other authors from measurements of PMMA fiber preforms [17] (see Figure 8). For this purpose, 50 m samples of both manufacturers were analyzed in an extended range of wavelengths, up to the near infrared region, where some of the data are available. It is clear that the results obtained in this work match satisfactorily with those reported from PMMA fiber preforms. Moreover, our calculated refractive indices also coincide with the values obtained from PMMA bulk material that can be found in the literature [18,25]. These similarities between our results and those reported from other measurement methods seem to corroborate the validity of our measurement technique and of our results. We would also like to emphasize that our measurement technique allows us to directly obtain the refractive indices and the coefficients of dispersion from the POF, without having to use the preform or the bulk material, which is the main novelty of our work.

## 5. Conclusions

We characterized both the refractive index and the coefficient of chromatic dispersion of PMMA POFs made by two different manufacturers. A tunable femtosecond laser and a Streak Camera were used for this purpose. The characterization technique is based on measuring the time delay between light pulses at different wavelengths when they propagate through POFs. Three different fiber lengths were considered for all the measurements. In all cases, very similar results were obtained, the maximum discrepancy between measurements being close to 3%. The obtained values match satisfactorily with previously reported values, which seems to corroborate both the validity of our measurement technique and our results. As far as we know, this is the first time that this kind of direct characterization from POFs has been carried out.

## Figures and Tables

**Table 1:** Main mechanical and optical characteristics of the fiber samples. PMMA = poly(methyl methacrylate); SI = step-index.

	Toray raytela	Mitsubishi eska
	PGU-CD1001-22-E	GH4001
Core material	PMMA	PMMA
Cladding material	Fluorinated polymer	Fluorinated polymer
Refractive-index profile	SI	SI
Core refractive index	1.49	1.49
Numerical aperture	0.5	0.5
Fiber diameter (mm)	1	1
Jacket diameter (mm)	2.2	2.2
Attenuation at 650 nm (dB/m)	≤0.15	≤0.19

**Table 2:** Coefficients *A* and *B* of Equation (3) together with the corresponding coefficient of determination of the fittings (*R*^2^).

	*A*	*B* (nm^2^)	*R*^2^
TORAY 25 m	1.470	5006	0.9917
TORAY 50 m	1.472	4907	0.9979
TORAY 75 m	1.473	4851	0.9976
MITSUBISHI 25 m	1.474	4707	0.9908
MITSUBISHI 50 m	1.476	4627	0.9943
MITSUBISHI 75 m	1.477	4580	0.9954
